# How Do Drug-Death-Bereaved Parents Adjust to Life Without the Deceased? A Qualitative Study

**DOI:** 10.1177/0030222820923168

**Published:** 2020-05-09

**Authors:** Kristine B. Titlestad, , Margaret Stroebe, Kari Dyregrov

**Affiliations:** 1 Faculty of Health and Social Sciences, Western Norway University of Applied Sciences, Bergen, Norway; 2 Department of Clinical Psychology, Utrecht University, Utrecht, the Netherlands

**Keywords:** grief, drug-death bereavement, parents, coping strategies, oscillation

## Abstract

Knowledge about how bereaved persons grieve can enhance quality in providing the support and potential services that they need. We aimed to identify ways in which drug-death-bereaved Norwegian parents go on with their lives and what inhibits or promotes adaptation during their grieving. Reflexive thematic analysis was used to analyze 14 semistructured in-depth interviews. We generated three themes: (I) processing grief emotions, (II) proactive coping, and (III) giving and receiving support and assistance. Processing guilt rumination, reflections on blame and a burden of grief emotions characterized grieving early on. Using cognitive strategies and functional-support-giving were found to be the most frequently used strategies. Oscillation between processing stressors and reorientation to the world promoted adjustment to ongoing life. We discuss characteristics of parents who struggle to reorient and outline important implications for policy and practice.

The way a person copes with the loss of a next of kin has been shown to impact on adaptation to bereavement (Nolen-Hoeksema et al., 2013, p. 17). Although grief is not a disease, and bereavement can be viewed as a normal experience, there is a risk of developing psychological and physical health problems after a loss of a loved one, especially after sudden, unnatural deaths (K. Dyregrov, 2003; Stroebe et al., 2007; Stroebe, Stroebe, et al., 2017). Drug-related deaths (DRDs) are unnatural deaths, ones that have reached epidemic proportions in the United States (21.7 per 100,000) (Centers for Disease Control and Prevention, 2019). They also present a major public health challenge across Europe (European Monitoring Centre for Drugs and Drug Addiction, 2019, p. 80). Clearly then, there is need for particular attention to the situation of bereaved family members left behind after DRDs as their unique difficulties have been identified in the scientific literature (see later).

The ability to identify grief reactions and to understand ways of coping are important, both for the bereaved and for professionals in the health and welfare services, as such knowledge can be used to enhance the bereaved person’s ability to adjust to life and to improve the services when these are needed (Crunk et al., 2019; Stroebe, Schut, et al., 2017). In this context, we think of coping as processes, strategies, or styles of managing the bereavement and define coping as the “changing thoughts and acts that an individual uses to manage the external or internal demands of stressful situations” (Folkman, 2001, p. 565). Grief reactions such as grief rumination and repetitive thinking about the causes and consequences of the loss and loss-related emotions can have negative impact on adjusting to life after a loss (Eisma & Stroebe, 2017). Such rumination is associated with depression, posttraumatic stress, and complicated grief (van der Houwen et al., 2010) and is a strong predictor of complicated grief (Eisma et al., 2013). By contrast, the construction of meaning has been identified as an important part of how people adapt to a traumatic death (Bonanno, 2013). The process of *meaning-making* may facilitate coping with the loss of a family member through a complex reorientation to the world (Neimeyer, 2019). Prior to using coping strategies, people might use proactive coping strategies that are “processes through which people anticipate or detect potential stressors” (i.e., what the bereaved have to deal with) “and act in advance to prevent them or to mute their impact (proactive coping)” (Aspinwall & Taylor, 1997, p. 417). Finally, social support has been identified as a major factor in coping. The extent to which parents experience support may relate to the degree that they adapt after the death (Greeff et al., 2011). K. Dyregrov and Dyregrov (2008, p. 50) found that bereaved ask for support from both their social network (e.g., family, friends, colleagues) and from other bereaved (peer support), in addition to professional help, as help and support from these sources address different types of needs. However, the time frame, amount, and needs of these different types will differ among grieving individuals and families (K. Dyregrov & Dyregrov, 2008). Bonanno and Burton (2013) also drew attention to the need for flexibility, emphasizing the importance of various strategies for ultimate adaptation. Such a concept would seem especially relevant in the case of DRD bereavement, given the range and complexity of associated stressors (cf. Templeton et al., 2017).

Several different theoretical models have been developed to help understand coping with bereavement (Stroebe, Schut, et al., 2017). One that explicitly captures flexibility in the coping process is Stroebe and Schut’s (1999) *Dual Process Model of coping with bereavement (DPM)*. According to this model, to cope effectively, a bereaved person must oscillate between *loss-orientation* and *restoration-orientation*. The former refers to coping processes that focus directly on the stress of the loss itself, including symptoms of grief, loss, and sadness, while the latter includes the processes one uses to cope with the secondary stressors (e.g., having lost one’s caregiving identity) that accompany one’s new status as a bereaved person (perhaps including symptoms relating to these changes, such as exhaustion and anxiety). Two additional features of the DPM, elaborated on more recently, are particularly relevant in the current context: (a) overload, “the bereaved person’s perception of having more than s/he feels able to deal with,” can affect the ability to oscillate (Stroebe & Schut, 2016, p. 100) and (b) family dynamics are understood to affect personal grief and vice versa (Stroebe & Schut, 2015).

Despite indications of its importance, given earlier, a systematic review by Titlestad, Lindeman, Lund, and Dyregrov (2019) showed that there has been hardly any investigation of ways that parents cope after DRDs. Yet, losing a person who used drugs is associated with stigma, complicated grief reactions, and unacknowledged grief (Titlestad et al., 2019; Valentine et al., 2016). Research evidence comes mainly from a qualitative substudy of 32 next of kin in England and Scotland (Templeton et al., 2017) and a quantitative study of 48 parents in United States (Feigelman et al., 2011). Templeton et al. (2017) described the stigma relating to DRDs, feelings of guilt, self-blame, and unworthiness to grieve. Feigelman et al. (2011) found a consistent pattern, whereby drug-death bereaved were consistently more troubled by grief and mental health difficulties than those bereaved after accidents or natural deaths. Adding to these, a qualitative study by Feigelman et al. (2020; *n *=* *11) reported how bereaved adjusted to life. Their findings showed that as time goes by, drug-death-bereaved parents “were able to arrive at a ‘new normal’ and reflect on their own posttraumatic growth (Feigelman et al., 2020, p. 17).” Taken together, the evidence so far suggests special difficulties and special needs among DRD-bereaved persons and a need to further explore how they cope and adapt to their loss and changed lives.

A large Norwegian study was launched in spring 2017. The purpose of the main project was to contribute to knowledge on bereaved family members and close friends after DRD. The main study was a mixed-method one, collecting quantitative data through a survey and qualitative data through interviews (ResearchGate, 2019). Definitions of DRD vary (Robertson, Bird, & McAuley, 2019). In this study we defined DRDs as deaths caused by the intake of substances classed as narcotics and deaths among people who use narcotics, where the cause of death is violence, accidents, infectious disease, and other health disorders, which in different ways may be linked to drug use. A distinction was made between drug- and alcohol-related deaths, and we focused only on drug death bereavement. Reactions to these two types of death likely differ. Using drugs is an illegal activity and, as Corrigan, Schomerus, and Smelson (2017) point out, the addiction stigma is likely to be worsened by criminalization, since drug use is conflated with felonious conduct. Moreover, people who die from an overdose are more likely to be male and young and to suffer a death that occurs early in the path of addiction (Templeton et al., 2017).

This article is one of two articles that explore drug-death bereaved Norwegian parents' grief. In the paper “Sounds of Silence” we reported how parents described a special death and how the silence from helpers, self-stigma and complicated interactions within the social network can contribute to a special grief (Titlestad, Mellingen, Stroebe, & Dyregrov, 2020).

The aim of the current article was to explore ways in which drug-death-bereaved parents go on with their lives after losing their child to drug use. Thus, the research question is as follows: “How do drug-death-bereaved parents adjust to life without the deceased and what seems to inhibit or promote adaptation during their grieving process?”

## Methods

To generate a phenomenological, hermeneutic understanding of how parents experience DRD, we used reflexive thematic analysis as described by Braun and Clarke (2019). We searched specifically for a variety of coping processes and strategies that are unique to drug-death bereavement. Semistructured in-depth interviews were carried out, and NVivo 12, qualitative data analysis software (QSR International Pty Ltd, 2018), was used in the data analysis process. This article was guided by “Standards for Reporting Qualitative Research: A Synthesis of Recommendations” by O’Brien et al. (2014).

### Recruitment and Sample Size

In the period from March 2018 until the end of December 2018, the main project enrolled drug-death-bereaved family members and friends by inviting them to fill in a questionnaire on paper or digitally. A flyer that described the project was sent to all Norwegian municipalities’ public email addresses. We also contacted personnel who were engaged in the Norwegian Directorate of Health project to reduce drug overdoses, involving 28 municipalities at that time. Recruitment was also facilitated through nongovernmental organizations working with drug use, treatment centers, the Norwegian Labor and Welfare Administration, and crisis teams (either by mail or by handing out flyers). We disseminated information about the project through participation at conferences and various media such as television, radio, and social media (Facebook and Twitter). *Snowball recruitment* by participants and by collaborators in other research networks or professionals in clinical practice was another important recruitment strategy employed.

The interview sample in this substudy was drawn from a total sample of parents. Ninety-five parents participated in the survey, and 75 parents agreed to be interviewed. Inclusion criteria were that the participants (a) had participated in the END-project survey, (b) spoke fluent Norwegian, and (c) had lost a child to DRD at least 3 months prior to recruitment. No other restrictions were set for the time since death. Because many more parents (*n *=* *75) agreed to be interviewed than could actually be included, the sample to be analyzed in this article was selected according to background variables such as gender, age, place of residency (city/village and northern/central/western/southern/eastern geographical part of Norway) of the parents, the time since death, and the age and gender of the deceased. Malterud et al. (2016) have proposed a set of considerations about different dimensions that influence the sample size such as the study’s aim, sample specificity, theoretical background, dialogue quality, and strategy for analysis. To ensure the adequacy of the final sample, we looked to Malterud et al. (2016) for their guidance on *information power*. After interviewing seven fathers and six mothers, we decided to equalize the sample according to gender, in case the descriptions specific to gender became relevant to our discussion. Another mother was therefore invited to participate. After interviewing her, and given that the contribution of new knowledge was limited, we concluded that we had reached a satisfactory level of information power.

### Semistructured In-Depth Interviews

A semistructured interview guide, built on the questions in the survey that we wanted to explore in detail, was developed for the interviews. The guide consisted of five themes: (a) the time before the death; (b) the loss; (c) stigma from the environment and self-stigma; (d) help, support, and coping; and (e) posttraumatic growth. In the preparation phase, the interviewing authors discussed subthemes that could possibly be relevant as follow-up questions in the interviews. During the interviews, we encouraged the participants to tell us about the deceased, their relationship to the deceased, the deceased’s living habits, the circumstances surrounding the death, their grief reactions and how the death affected their health, working situation, and leisure time. In relation to stigma, we talked about attitudes emanating from their surroundings (family, friends, work colleges, etc.) and how they and others in their network communicated about the loss. We encouraged the parents to reflect on support from family, friends, colleagues, social networks, and support groups and help from health and social services, the police, ambulance personnel, priests, crisis teams, and so forth. We also asked the participants to share their thoughts about potential barriers to support and what help and support they needed, in addition to barriers or facilitators of own coping and meaning-making. However, first and foremost, the interview method required the researchers to pursue the thoughts and reflections of the interviewees. The interviews were carried out in the period August 27 to December 4, 2018. To synchronize the interview method and pilot test the interview guide, the last author conducted a trial interview with a bereaved parent, with the first and second authors (interviewers) present. The interview was discussed with the bereaved and the research interviewers. The interview guide was adjusted according to discussions after the trial interview and prior to other in-depth interviews.

Following completion of the informed consent process, the first author conducted six, the last conducted five, and the psychologist three in-person interviews in a private setting selected by the participant (home = 9, work office = 4, a hotel [sheltered space] =1). The interviews were audiotaped and transcribed verbatim by a research assistant. In addition, all interviewers noted their general impressions immediately after each interview. The length of the interviews ranged from 1 hour and 20 minutes to 3 hours and 10 minutes, including required or desired breaks. Altogether, the transcripts consisted of 431 single-spaced pages (range 20–39).

### Sample

The sample consisted of 14 parents: 7 women and 7 men, who were parents to 14 deceased persons in total. One mother withdrew for personal reasons and one of the recruited participants failed to attend the planned interview. We were unable to reach out to the latter individual, either during or after the interview time, and no explanation was given as to why the potential participant decided not to keep the preplanned appointment.

One parent represented two deceased people, and a divorced couple represented one deceased person. All the parents were aware of the drug use, and none of the deceased had died after first-time use. Ten of the parents had lost a son, and four parents had lost a daughter. The time since death ranged from 3 to 126 months (*M* = 38), and all parents reported (on a 5-point Likert scale) to have been close to the deceased (12 reported to have been very close). The age of the deceased varied between 19 and 45 years (*M* = 27.36), and the age of the parents ranged between 45 and 75 years (*M* = 58.29). The participants reported to have experienced, on average, three demanding life loads. They came from all parts of Norway (north, *n *=* *2; central, *n *=* *2; west, *n *=* *5; south, *n *=* *2; and east, *n *=* *3), with eight living in a village and six in a town. Twelve of the parents were married/cohabitants, while one had a boyfriend, and one was divorced. Only 2 of the 14 participants were still married to the other parent of the deceased. The parents were well educated (79% had received higher education beyond 12 years). Annual household gross income was in the range from 28,500 to more than 143,000 dollars, and 42.9% had an annual household gross income of 85,500 to 114,500 dollars. The income level in Norway is high, and the participants’ income was high compared with the average annual household gross income.

### Reflexive Thematic Analysis of Interviews

Braun and Clarke (2019) describe a six-phase process for reflexive thematic analysis: (a) familiarization with the data, (b) coding, (c) generating initial themes, (d) reviewing themes, (e) defining and naming themes, and (f) writing up. The phases are sequential, each build on the previous one, and the analysis is therefore a recursive process. We analyzed the interviews as recommended by Braun and Clarke (2019), conducting a reflexive thematic analysis with movement back and forth between different phases.

After reading and rereading all the interviews to become immersed and intimately familiar with their content and coding the entire data set in NVivo, we examined the codes and collated data to identify significantly broader patterns of meaning (potential themes). Themes, defined as patterns of shared meaning underpinned by a central concept or idea (Braun & Clarke, 2019), were then defined. Moving back and forth between the phases, clustering of themes was generated. We worked out the scope and focus of each theme, deciding on an informative name. A table of the codes and themes was then produced.

The analyses were conducted by the first author (a social educator) in collaboration with the last author (a sociologist) and then altered in accordance with consensus discussions with the second author (a psychologists). Trustworthiness of the findings was enhanced by thorough discussions among the authors. All authors agreed upon the coding framework, the interpretation of the data, and the confirmation of codes and themes.

### Ethical Considerations

All procedures were conducted in accordance with the Declaration of Helsinki (The World Medical Association, 2018). This study was approved in February 2018 by the Norwegian Regional Committees for Medical and Health Research Ethics (reference number 2017/2486/REK vest).

All participants signed a written informed consent form that described the purpose, method, and procedure of the study, and the participants were informed that the data would be published in a nonidentifiable manner. Care was provided to the participants during the entire interview process according to K. Dyregrov’s (2004) recommendations concerning research on vulnerable populations. The parents were assured of anonymity, confidentiality, and the option to withdraw from the study at any time. The interview data were treated confidentially. All identifying information concerning transcripts and recordings was de-identified and stored in the research server at the university.

## Results

**Figure 1.**
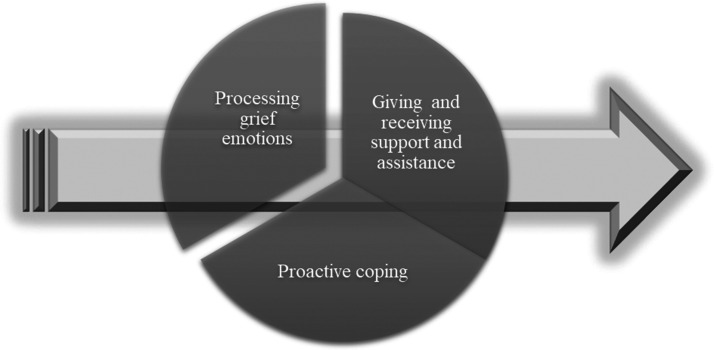
Three Interconnected Themes Describe How Drug-Death-Bereaved Parents Adjust to Life Without the Deceased.

The mothers and fathers each told their own story, sharing their individual experience and personal perspective. How they adjusted and adapted to life without the deceased were influenced by both intrapersonal and interpersonal management strategies, as well as by internal and external circumstances. Even though they had adjusted to life in varying degrees and in varying ways, there were three interconnected themes that were identified from the data: (I) *processing grief emotions*, (II) *proactive coping*, and (III) *giving and receiving support and assistance* (Figure 1).

As would be expected, losing a child triggered many negative emotions. Repetitive and recurrent thinking regarding guilt was the most striking negative emotion reported by the parents. This processing of grief emotions was connected to three codes: (a) ruminating about guilt, (b) reflections on blaming others, and (c) adaption to external triggers.

### Processing Grief Emotions

As would be expected, losing a child triggered many negative emotions. Repetitive and recurrent thinking regarding guilt was the most striking negative emotion reported by the parents. This processing of grief emotions was connected to three codes: (a) ruminating about guilt, (b) reflections on blaming others, and (c) adaption to external triggers.

#### Ruminating About Guilt

Many of the bereaved had ruminating thoughts before the death. They felt guilt for not making the right parental decisions early in their child’s life and blamed themselves for the predicament their child had been in. When the child died, and all hope was gone, rumination associated with guilt escalated. The bereaved ruminated about why they had failed to prevent their child from using narcotics, hanging around with *the wrong* friends, and for working away from home. They ruminated about whether they should have used more punishing strategies, yet at the same time, they were convinced that putting their child *in chains* would not have solved anything. A mother who had lost her son 18 months previously ruminated as follows: “Why? Why, why couldn’t we? Why couldn’t we stop him, and should we have done something different? Should we have punished him, been stricter? These thoughts arise, were we stupid, blind?” (ID 15)

For many of the parents, guilt rumination decreased, but the extent and persistence over time after the loss differed from parent to parent. The parents described how they had become reconciled to the fact that they had done their best to help their child and to prevent the death, as expressed by one mother:

So I have no guilt today, because I know I’ve done everything I could. I couldn’t do any more. I might have done things differently, some of it, but no, I really couldn’t. Well, he always knew that I was there for him and that he could always call me. (ID 7)

Although the parents explained that they had learned to live with the guilt, some also pointed out that guilt rumination was seething in the back of their mind and that guilt and negative thoughts could arise when they least expected it.

#### Reflections on Blaming Others

The bereaved reported that they had many questions about whether the death was an accident, suicide, or if their child was killed. They wanted answers about what part others played in their child’s addiction to narcotics and why the child did not get the help she or he needed. This father reflected that bitterness could easily be the outcome of ruminating over who was responsible:

After the loss, I could very well have gone into a state of bitterness (mhm), and of course at times, there were lots of questions and critical issues. I never got straight answers, though when you are in this situation, right after the loss, you try to get answers to a lot of things, but I do not think there were clear answers. Still, the bitterness - nothing good comes from it. (ID 26)

The parents often blamed the deceased’s friends. As for this father, a coping strategy for many of the bereaved was that they accepted that nothing good came out of blaming others. That many of the deceased prior to their death had encouraged them to stop blaming others and helped them put aside this rumination. After some time with rumination about who to blame, many reported that they acknowledged that their child’s friends struggled just as much as their child. All the parents, except one, said that the loss had changed their way of thinking. Some said they had changed perspective regarding what was important in life, they were softer and tougher at the same time, while others said that they were more tolerant toward other people’s challenges.

The parents also blamed those working in public services, health services in particular. It was harder for the parents to comprehend and accept that services did not provide appropriate health services that could have kept the child alive or that general practitioners (GPs) prescribed drugs that, in their opinion, their children were not in need of.

#### Adaption to External Triggers

Many of the parents experienced grief emotions that were triggered by external circumstances. Overwhelming sorrow, such as bursting into tears or experiencing anxiety, could be triggered by places they associated with the deceased or through watching a movie that brought back memories. Holidays and memorials also brought on grief reactions with unexpected waves of emotions. Like this father, several bereaved described listening to music to be a trigger:

Yeah, well, it didn’t take much to trigger me for quite some time, though I simply thought of it as a relief. And that I got proof for myself of how much I cared, so it was, (that is good to hear), it was actually a good, a decent feeling in a way. (ID 26)

This father also described that he used music therapeutically in the grief process. Over time, most of the parents experienced that the impact of triggers of complex emotions decreased or triggered different emotions. Most of the parents reported that the reactions got easier to cope with, and as time went by, the emotions were not so overwhelming. Some triggers changed from initiating grief reactions to acting as a coping mechanism. Listening to music could eventually calm the father mentioned previously, and a mother experienced that after some time, it calmed her down when sleeping in her daughter’s bed at their family cottage, contrary to the distress she experienced when visiting the cottage right after the loss. On the other hand, some parents actively avoided some triggers, especially locations they knew that would trigger reactions, such as places they associated with the deceased.

### Proactive Coping

As time goes by, most of the parents adjusted gradually to life without the deceased. Four codes reflect active sets of strategies: (a) cognitive strategies, (b) communication strategies, (c) craving knowledge, and (d) back to day-to-day activities.

#### Cognitive Strategies

All parents described that they worked with their *mindsets* by using cognitive strategies to deal with complex emotions and reactions. Many parents described this as hard work that required great will, reflection, and pragmatism. They had to change their way of thinking. For example, those who experienced existential thoughts stated that taking control of their mindsets was vital. One father called it a mental defense wall and explained how he took control by talking to himself, that he gave himself instructions on how to react and behave. In this way, many of the parents decided to make room for grief. This mother’s strategy was to acknowledge that she had bad days and to allow herself time-outs for grieving:

I am very good at allowing myself to have bad days and feel the loss, and they come less frequently now, but they came much more often before. And then I thought, then it was just like, it is of no use to try today, today is just one of those days … use strategies I found are good for me … not hold back, … So you lock yourself inside on Friday and go back out again on Monday morning, because you just have to disconnect from the whole world and everything, right.  … I’ve done that very consciously; I’ve worked hard to move on. (ID 125)

In addition to deciding when to grieve, several of the parents also decided to put aside grief. A father described the oscillation like this:

I had to … really listen to my feelings, to the extent that I felt it and endured it. And tolerating the grief, eh, doing what I felt I could manage, no more, stay still, but also be active … . I can’t be grieving all the time, so I think that has worked for me, sort of switching between sadness and a totally, totally OK feeling. Not jumping for joy, but like I’m fine and I can enjoy things too. (ID 57)

However, some experienced that putting emotions aside also had serious consequences. Strategies that at one point felt like a coping strategy turned into an avoidance mechanism, and over time putting emotions aside made it all worse. Most parents experienced that they were better able to bear their grief as time went by, though many described that, despite trying hard to control their emotions, a wave of grief emotions could overwhelm them, without any warning.

#### Communication Strategies

Almost all the parents were open to others about why their child had died. This category of communication strategies reflects how parents actively chose with whom they wanted to communicate about the loss. Again, the bereaved preferences differed, but all experienced that talking was therapeutic, a coping strategy used to aid acceptance of the loss, and that they often repeated themselves, even to the same listener. A mother described how she also used taking about the loss to cope with self-perceived stigma:

It is probably more like you are looking for it, that is, you feel it so strongly, that everyone thinks it is your fault. If you had managed to take better care of your daughter, then this would not have happened, everyone thinks so. So you see it in people’s body language, their eyes, or hear it in things they may not even say. Eh, so for me, I had to be proactive, it became sort of important to me not to, I would rather be open about it. … “Yes, she died of an overdose, yes, she died of intoxication, no, she did not have it easy and I have done my best.” (ID 62)

For many of the parents, it was also important to talk about the deceased to honor the person’s memory, fearing that others would forget him or her. As they felt that communicating about the child was not that easy for all in their network, the bereaved had preferences toward whom they preferred to talk to, more often close family members or a few close friends.

#### Craving Knowledge

Some of the deceased persons had trouble adapting to the expectations from, for example, the school system, and some had mental health problems. Growing up, most of the deceased had experienced challenging life events such as sexual abuse. The parents described that they ruminated about the extent to which genetic factors or certain life events caused their child’s addiction to narcotics. Several of the parents called on skilled personnel to learn more about drug addiction and/or sought out professionals who had treated their child. Like others, new knowledge helped this father to cope with and accept the loss:

Reason, I’m probably never going to find the whole reason, but just to understand in a way what happened to him [the deceased]? Getting a better understanding of what happened would have been nice, what happened to him [the deceased], that’s really my grief process. I’ve come a long way, and I will never quite see the end of this, these questions, but I have to understand a little more about the effects of drugs and drug use and, yes, just hear people talk about similar cases and say that this is completely normal.  … Well, it would be like, settling my mind. (ID 42)

Even though all the parents feared losing their child, it is a devastating shock to many of them when death occurs. Many ruminated about what happened during the time leading up to the death, and the parents wanted to know who was involved or present at the time of the death. The parents therefore sought out to police, health-care professionals, and GPs who had been in contact with their child. The parents, who used this information to make a time line for the death event, described that this knowledge reduced their rumination, even though not all their questions were answered.

#### Back to Day-to-Day Activities

Many of the bereaved used their energy after the loss to deal with practical tasks necessary after a death. A plan for the day, and going through with this plan, kept many parents on their feet in the time immediately after the death. Returning to work was described as a very important action to adjust to life without the deceased. For many, as this father described, going back to work was the most important action after the loss:

 … the most important thing I have done to move on after the death was that I was able to go back to work, in light of returning to the community you are supposed to be in, or daily routine in a way, getting the machinery running again. (ID 96)

Flexible and concerned employers and colleagues were important to stay in work. The parents praised their GPs especially, for using graded sick leaves and making a back-to-work plan. Bereaved, who did not have an employer or were not part of a positive work environment, stated that they struggled more, both with finding meaning with work and with life. Other activities that were described as important managing strategies were hiking, jogging, knitting, and meditation.

### Giving and Receiving Support and Assistance

This theme reflects on external circumstances that parents experienced which strengthened their ability to adjust to life, as well as covering descriptions of reactions to lack of help. Four codes were identified: (a) to be needed by others, (b) social network support, (c) professional assistance, and (d) peer support.

#### To be Needed by Others

The parents described how they pulled themselves together after the loss in consideration for the deceased’s sibling(s) or child(ren). Most of the parents cared for, and some took over the parental responsibility for, the bereaved grandchild(ren). Even though some said being the main care provider at times was difficult, taking care of others helped parents cope. Functional-support-giving prevented this father from collapsing:

But moving on is a must, I definitely have to do that, and I have to be, I must also be, strong and ready for my daughter [the deceased’s sister] who needs a father who is there for her, and who can support her, so I can’t break down. (ID 42)

Some parents describe being needed by others as the most important factor for getting back on their feet and that caring for others was the main motivation for going on with their lives. Their children were also a bright spot in the bereaved parents’ lives, and the parents found strength in fighting to prevent that the sibling or the deceased’s child from walking in the same footsteps as the deceased. Nevertheless, parental responsibility also limited their grief; they did not allow themselves to show their child(ren) how the loss affected them. Being responsible for other children or grandchildren also strengthened cohesion in these families. A mother described how sharing this experience seemed to reinforce positive family dynamics and reduced and relieved their own pain (ID 15).

Parents who had lost their only child or did not have other children who depended on them struggled more to adjust to life. After several years, with the child who continuously needed help and care, one father described that not to be needed was like being hit by a meteorite strike; the landscape had completely changed, and all the reference points were gone. “It is very difficult to find something to take a compass course, because you have nothing to put it on,” he said (ID 39).

#### Social Network Support

Two of the 14 bereaved parents were still married to the childs other parent when the child died; five parents described ongoing conflicts with their former spouses, while others described a good collaboration. Experiencing the loss did, in most cases, strengthen the ties between family members, but those who were already in tough conflict prior to the loss experienced that the death worsened the conflict.

Many bereaved described supports by family members, friends, and extended network immediately after the death, and parents found comfort in an unexpected support from the child’s friends. This mother was clearly affected when she described how much the support meant to her:

(her voice cracking with emotions) The days were hectic, but I actually experience a lot of support, there are people here all the time. I don’t really know how to relate to all the people, but I actually think it’s okay that they are here. Then some leave, some stay, and some come and go again, and I receive flowers and cards and it keeps on coming.  … And at the funeral the friends could come in first.  … I sat out of sight. I couldn’t bear their grief. But there is something about adolescents that differ from the adults who are more enclosed and (sigh) afraid to say something wrong. These youths, they were crying, and they were asking, and they were talking, and they were hugging each other and hugging me and wanted me to sit with them (voice cracking with emotions). So we were kind of together, and I remember that like … (crying). (ID 62)

They described how people in their network fulfilled different needs. Family members were their most important support in grieving over the loss, while close friends were important in the way they provided free space that they needed to put their minds on other things. Some of the bereaved pointed out the importance of telling others what you need, as it is difficult for others to understand and know. Importantly, parents described close networks as the most helpful in the process of adjusting to life. However, over time their networks narrowed down to a few very close family members and friends, as some bereaved found it difficult to communicate with people in their social network, especially those who lacked knowledge about addiction. Some also felt that others did not seem to handle bearing their grief and therefore did not talk about the deceased or ask how the bereaved was doing. Many bereaved coped with this behavior by excusing these people, saying that they themselves before the loss could have expressed ignorant statements about addiction and that they probably would not have known how to give support.

#### Professional Assistance

All parents expressed a need for professional help after the loss, and yet only 1 of the 14 interviewed parents was contacted by a local crisis team. Most of them described that they had to take the initiative and reach out to get the help they needed from public services, and even when they reached out, some felt neglected/ignored. Several of the parents received help from the deceased child’s therapists, but most parents said that they reached out to a GP who referred the bereaved to a specialist (e.g., a psychologist, grief therapist) or to a grief group.

Eventually most of the parents got help, though some experienced that their GPs requests (e.g., for them to receive therapy) were rejected. Those who received help expressed that the assistance was of good use. They learned strategies to cope both with the emotional burden and self-perceived guilt, as well as coping strategies for day-to-day living. This mother described how she also got help to understand her grief reactions:

So the best thing about that psychologist (light laughter in her voice), he was absolutely fantastic at explaining to me how the brain works and he could explain to me why I reacted the way I did. Because I, I didn’t know, I never thought I could be depressed (no), it was far beyond my thoughts. Of course, when I think back, I reflect on why haven’t you been depressed long ago? (laughter) (yes). But at that time, it was not even in my mind, but now I am careful not to relapse. So, just as I approach the edge, then I know I must do something, and that is what he has taught me. (ID 7)

#### Peer Support

The few parents who were referred to a support group described that the threshold for taking in the group was low and that there was a basic understanding about what it is like to lose a child. Talking to people who had the same experiences helped, though some parents needed breaks from the group like this father:

And that has helped me many times, that I have been able to talk to others, or listen to others. Maybe comforted others when I have come a little further … . And there are often friends and others who motivate me or ask me to talk to others. Then for a while it was a bit too much. Although, it somehow became something you can give back, that helps me move on. (ID 125)

For the parents, it was important that the other bereaved in their support group had experienced DRD. They argued that it is impossible to fully understand drug-death bereavement if you have not experienced it yourself. Those who participated in grief groups with bereaved after other causes of death also argued that support groups for drug-death bereaved would be more beneficial.

## Discussion

The themes (I) *processing grief emotions*, (II) *proactive coping*, and (III) *giving and receiving support and assistance* describe how drug-death-bereaved parents adjust to life without their child. The three themes are intertwined and were shown to influence each other; together, they helped to describe what inhibits or promotes adjustment to life after DRD loss. Also, the themes aid understanding of coping with bereavement as a personal as well as an interactional pathway. The codes (IIa) *cognitive strategies* and (IIIa) *to be needed by others* comprise the main findings promoting adaption, while (Ia), *Ruminating about guilt*, dominated the parents’ descriptions of reactions that had a negative impact on adjusting to life after the death. Adjusting to life was mainly described through oscillating between stressors, functional-support-giving, and proactive coping. Challenging communication was reported to be a hindrance, and the bereaved parents called for help from services and support, and from peers.

### Adjusting Through Oscillating Between Stressors, Functional-Support-Giving, and Proactive Coping

**Figure 2.**
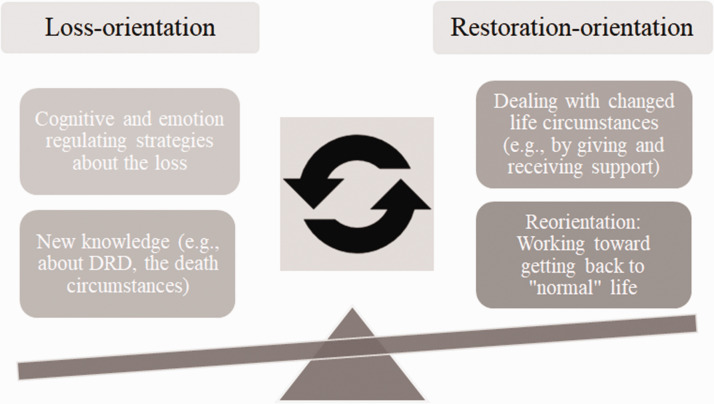
Illustration of How the Parents Oscillated Between LO and RO Strategies as They Adjusted to Life. DRD = drug-related death.

The findings of our study illustrate certain parameters of the DPM, placing our results in theoretical context. Loss-orientation coping, such as rumination and using cognitive and emotion regulating strategies, characterized the first time period after the loss (Figure 2). Restoration-orientation coping that actually increased the sense of the loss was also found in this study; changed roles in relation to the deceased child(ren) was the most striking finding. The participants reported oscillating between processes such as actively grieving (loss-orientation) and going back to day-to-day activities, such as, in some cases, returning to work (restoration-orientation). These findings of alternation between stressors and other activities illustrate the oscillation process as an integral part of the process of coping with grief and grieving. The results resonate with those of a parental bereavement study by Albuquerque et al. (2017), which highlighted the importance of being flexible. In line with Bonanno and Burton (2013), these findings show the importance of coping and emotional regulation as flexible adaptation, stressing that responses are rarely static. There are reasons to argue, then, that, on a personal level, flexible adaptation and oscillation between loss-orientation and restoration-orientation processes promotes adjustment to life for many of our grieving parents.

Our study showed how important the restoration-orientation strategy incorporating distraction from the loss itself was, notably, by taking care of the other children; those who had lost their only child and/or did not have other children who depended on them struggle more to adjust. In our view, these results illustrate that restoration-orientation stressors are important to deal with (e.g., finding a new role represents a needed reorientation to the world). We also conclude that functional-support-giving was also an important part of the meaning-making process that facilitated coping with the loss. In the DPM-Revised, Stroebe and Schut (2015) recognized how family dynamics can impact individual adjustment in either positive or negative ways. We argue that the family-level stressor of being needed by others actually promoted adjustment, while ongoing conflicts with former spouses inhibited the bereaved person’s grieving process.

Extensive rumination, combined with the generally more adaptive *working through* grief (cf. Stroebe, Schut, et al., 2017), was one of the most striking results in our study. Most parents described how it helped them not only to ruminate about their loss but also to process grief emotions, especially self-perceived guilt and blaming others. Oscillating between maladaptive rumination and eventually more and more adaptive cognitive processing helped many parents set aside guilt and the blaming of others. Stroebe and Schut (2016) argued that coping with bereavement according to the DPM is a complex regulatory process of confrontation and avoidance. Our study’s findings suggest that avoidance as well as confrontation was an important coping mechanism; for example, for longer term adjustment, it seemed to be necessary to oscillate between loss-orientation and restoration-orientation (as well as take time off from coping) to avoid a resurgence of grief.

The stressors, particularly those relating to guilt rumination, were at times difficult to deal with and at times too much for the bereaved to handle. According to Stroebe and Schut (2016), overload can hinder the bereaved person’s ability to oscillate *effectively* between loss-orientation stressors on one hand and restoration-orientation ones on the other hand. The finding in our study is consistent with this; the parents who described an extensive overload after the loss were the ones who had most difficulties with flexible adaption and oscillation. These parents also described a need to put the overload aside to ignore the stressful things that needed to be coped with, which in the long run made reorienting even more difficult.

The parents' descriptions of grief following DRD is reported in Sounds of Silence. The Special Grief of Drug-death Bereaved Parents (Titlestad et al., 2020). In that article we discuss how the time before death imposed a considerable emotional and practical burden on these parents, one that causes a special grief. Folkman (2001, p. 564) pointed out that coping may sometimes have less influence on adjustment compared to factors such as the timing and nature of the death, history and personality. Therefore, we argue that the drug-death bereaved parents' history, as well as the nature of the child's death, must be taken into consideration when DRD bereaved parents need assistance and support.

Many parents described that they used proactive coping strategies, and cognitive strategies were the most frequently used of these. For example, deciding to go back to work, making room for grief, and ceasing to blame others probably also helped to reduce overload. Deciding at times simply not to confront one’s grief also served to bring some respite. The bereaved participants reported not only that avoidance inhibited adjustment but also that alternating between *making room* for grief and taking time off grief was an important coping strategy. Furthermore, proactively avoiding some of the triggers, such as places they associated with the deceased, was always described as an adaptive strategy.

### Challenging Communication and Call for Peer-Support Groups

This study shows that the parents who seemed to have the best interactions were the ones who were proactive, who clearly stated how and when they wanted to communicate about the loss. Even though the parents used *ventilating* as a strategy to cope, as described by K. Dyregrov and Dyregrov (2008, pp. 38, 47), the parents undercommunicated their grief because they experienced that other people seemed helpless with regard to talking to them about the DRD loss. The term *social ineptitude* has been used to explain the withdrawal of network members, unsuccessful contact, and communication problems as experienced by traumatically bereaved (K. Dyregrov, 2006). Openness, an assertion of personal needs, involves educating others as to how to support them (K. Dyregrov & Dyregrov, 2008, p. 118) and can help the bereaved deal with the overload (Stroebe & Schut, 2016). In line with other studies of unnatural deaths (K. Dyregrov & Dyregrov, 2008; K. Dyregrov et al., 2018; Feigelman et al., 2020), there are good reasons to argue here that proactive bereaved persons can help people in their social networks to take the perhaps difficult step to talk about DRD. Social networks therefore need to be informed of their potential role through listening with respect and through empathy.

Our findings showed that families with good dynamics before the loss shared their grief and were brought closer together. The loss of a close family member has been shown to affect family interaction (Stroebe et al., 2013) and also create a need for reorganizing the family structure (A. Dyregrov & Dyregrov, 2015; Stroebe & Schut, 2015). A. Dyregrov and Dyregrov (2015) studied bereaved parents’ perceptions of their relationship following the loss of a child. Our study supports their findings that talking together and communicating thoughts and feelings aid adjustment on a family level. Many divorced parents in our study were in conflict before the death; lack of mutual understanding and respect between former partners seems likely to hinder adjustment and complicate their relationships after the loss.

However, only a few of the participants in our study were offered participation in support groups. The parents stated that talking to other bereaved who had experienced DRD could be an important coping strategy and called for efforts to organize support groups specifically for drug-death bereaved. Bartone et al. (2019) synthesized studies regarding benefits of peer-support services for bereaved of sudden or unexpected deaths. These authors showed that peer support is beneficial in reducing grief symptoms and increasing well-being and personal growth. Peer support appears to be especially valuable for bereaved following suicide loss, a result that may be related to stigma. In addition, the study by Feigelman et al. (2020) of drug-death-bereaved parents, helping others by facilitating support was described as an important meaning-making strategy. Whether or how drug-death-bereaved parents benefit from facilitating and/or participate in peer-support groups is therefore an important topic for further investigation.

### Methodological Issues

To improve methodological rigor, we have restoration-orientation a transparent and clear description of the research process from the initial outline through the development of the methods and the reporting of the findings. Reflexive journals were written after each in-depth interview, containing information about our subjective responses to the setting and the participants. In addition, the results section contains key, illustrative, verbatim extracts from the interviews. Describing reflexivity is important to enhance a study’s validity. As recommended in standards for reporting qualitative research (Malterud, 2001; O’Brien et al., 2014), we described the characteristics and the role of the researchers in the article. We argue that the checklist for reporting standards strengthens the transparency of this study and enhances the transferability of its findings to other contexts.

One strength of this study is the wide use of different recruitment strategies. Nevertheless, despite our efforts to recruit bereaved parents from all classes in society, the risk of sampling bias is present, particularly given that people from lower social classes are underrepresented. On the other hand, the size of our study sample has sufficient information power, in accordance with Malterud et al. (2016) descriptions.

## Conclusion and Implications for Practice and Policy

The aim of the present research was to examine how drug-death-bereaved parents adjusted to life without their deceased child and what seemed to inhibit or promote adaptation during their grieving process. The parents essentially helped themselves using proactive coping strategies, and the family stressor of being needed by others was described as the most important factor in the meaning-making process. They oscillated between loss-orientation and restoration-orientation strategies, and flexibility between different strategies seemed to promote adaption. There were parents in this study who still struggled with reorientation to the new life circumstances. There is a need to pay special attention to the parents who experience an enduring overload before and after the death, to those who ruminate persistently about their own guilt, and finally, to those who describe their grief process as one of being stuck in either rumination or avoidance.

There is call for attention to dissemination of better knowledge about DRD, to reduce perceived stigma and facilitate peer-support groups for drug-death bereaved. The bereaved themselves need increased knowledge about addiction to narcotics to help them understand what happened to their child. All the parents, except one, had to ask for help, and some were even not given it. Those who got help emphasized that individual therapy promoted coping with the loss. In therapy, they learned how to understand grief reactions and strategies to cope with the emotional burden, self-perceived guilt as well as coping strategies for day-to-day living. The Norwegian white paper *The Psychosocial Interventions in the event of Crisis, Accidents and Disasters* recommends action plans for follow-up in the municipalities after a sudden and potentially traumatic death (Norwegian Directorate of Health, 2016, pp. 31–35). The main features of the plans are early and active outreach, broad spectra and adapted help for all family members, and help over time. Help from health and social services based on existing guidelines needs to be readily available to avoid intense grief reactions and promote adaptive coping strategies.
